# Low sodium intake ameliorates hypertension and left ventricular hypertrophy in mice with primary aldosteronism

**DOI:** 10.3389/fphys.2023.1136574

**Published:** 2023-02-15

**Authors:** Zitian Wang, Xue Zhao, Lifang Bu, Kun Liu, Ziping Li, Huaxing Zhang, Xiaoguang Zhang, Fang Yuan, Sheng Wang, Zan Guo, Luo Shi

**Affiliations:** ^1^ Department of Neurobiology, Hebei Medical University, Shijiazhuang, Hebei, China; ^2^ Department of Laboratory Medicine, Hebei University of Chinese Medicine, Shijiazhuang, China; ^3^ Core Facilities and Centers, Institute of Medicine and Health, Hebei Medical University, Shijiazhuang, Hebei, China; ^4^ Hebei Key Laboratory of Neurophysiology, Shijiazhuang, Hebei, China

**Keywords:** primary aldosteronism, hypertension, left ventricular hypertrophy, low sodium, myocardial metabolic

## Abstract

The goal of this paper is to elucidate the effects of sodium restriction on hypertension and left ventricular (LV) hypertrophy in a mouse model with primary aldosteronism (PA). Mice with genetic deletion of TWIK-related acid-sensitive K (TASK)-1 and TASK-3 channels (TASK^−/−^) were used as the animal model of PA. Parameters of the LV were assessed using echocardiography and histomorphology analysis. Untargeted metabolomics analysis was conducted to reveal the mechanisms underlying the hypertrophic changes in the TASK^−/−^ mice. The TASK^−/−^ adult male mice exhibited the hallmarks of PA, including hypertension, hyperaldosteronism, hypernatremia, hypokalemia, and mild acid-base balance disorders. Two weeks of low sodium intake significantly reduced the 24-h average systolic and diastolic BP in TASK^−/−^ but not TASK^+/+^ mice. In addition, TASK^−/−^ mice showed increasing LV hypertrophy with age, and 2 weeks of the low-sodium diet significantly reversed the increased BP and LV wall thickness in adult TASK^−/−^ mice. Furthermore, a low-sodium diet beginning at 4 weeks of age protected TASK^−/−^ mice from LV hypertrophy at 8–12 weeks of age. Untargeted metabolomics demonstrated that the disturbances in heart metabolism in the TASK^−/−^ mice (e.g., Glutathione metabolism; biosynthesis of unsaturated fatty acids; amino sugar and nucleotide sugar metabolism; pantothenate and CoA biosynthesis; D-glutamine and D-glutamate metabolism), some of which were reversed after sodium restriction, might be involved in the development of LV hypertrophy. In conclusion, adult male TASK^−/−^ mice exhibit spontaneous hypertension and LV hypertrophy, which are ameliorated by a low-sodium intake.

## 1 Introduction

Primary aldosteronism (PA) has been implicated in increased cardiovascular morbidity (Zennaro et al., 2020) and is a common cause of secondary hypertension, with a prevalence ranging from 5% to 10% among hypertensive patients (Funder et al., 2016). In addition, hyperaldosteronism is an independent risk factor for cardiovascular disease (Milliez et al., 2005; Monticone et al., 2018). Although studies have emphasized the negative role of aldosterone in left ventricular (LV) hypertrophy, the mechanism by which hyperaldosteronism modifies myocardial hypertrophy remains unexplored.

Experimental evidence suggests that excessive aldosterone-induced cardiac damage strictly requires a high sodium environment (Brilla and Weber, 1992a; Martinez et al., 2002; Rocha et al., 2002). This notion is strongly supported by a study of the New Guinea hill tribes, and who typically eat a very low salt diet (2–3 mEq/day) and have extraordinarily high plasma and urinary aldosterone levels but with normal blood pressure (BP) and no cardiovascular damage (Vaidya et al., 2018). However, due to the lack of appropriate animal models and technical limitations, the relationship between salt status and aldosterone-induced myocardial hypertrophy in PA still needs further elucidation. In addition, as a result of the heart’s relentless need for energy, most cardiovascular diseases are caused by disturbances in cardiac metabolism, and cardiometabolic characteristics in patients with PA remain unclear.

The PA mouse model used in this study was first established in 2007 by Davies et al. (2008). This mouse model was derived from the global deletion of TWIK-related acid-sensitive K (TASK)-1 and TASK-3 channels. TASK-1 and TASK-3 are two-pore domain K channels that contribute to negative membrane voltage changes by forming background or “leak” K channels (Goldstein et al., 2001). Male mice lacking TASK-1 and TASK-3 genes (TASK^−/−^) exhibit significant membrane potential depolarizations in their adrenal zona glomerulosa cells, resulting in autonomous aldosterone production (Davies et al., 2008). TASK^−/−^ mice also exhibit low renin activity, hypertension, and either the dietary sodium loading or the administration of angiotensin receptor blocker failed suppress aldosterone production. Thus, such mice are an appropriate choice for testing the hypothesis that equivalently raised (or even higher) levels of plasma aldosterone in the presence of chronic sodium deficiency do not cause cardiovascular damage. In this study, we first evaluated the cardiac structure and function of such TASK^−/−^ mice and then observed the effect of sodium restriction on hypertension and LV hypertrophy. In addition, we used untargeted metabolomics to predict the possible metabolic mechanism of hyperaldosteronism-related LV hypertrophy in the TASK^−/−^ mice.

## 2 Materials and methods

### 2.1 Animal grouping and treatment

Wild-type C57BL/6 mice (TASK^+/+^ mice) were purchased from Beijing Vital River Laboratory Animal Technology (Beijing, China). PA model mice (TASK^−/−^ mice) were kindly gifted by Dr. Douglas Bayliss from the University of Virginia (Davies et al., 2008). The animals were housed at the Laboratory Animal Center of Hebei Medical University and maintained on a 12-h light/dark cycle (7:00–19:00 light, 19:00–7:00 dark), with constant room temperature (22°C ± 1°C), and humidity (50% ± 10%) and free access to food and water.

To observe the effects of a low-sodium diet (LSD) on hypertension and myocardial hypertrophy in adult TASK^−/−^ mice, 13-week-old male TASK^+/+^ and TASK^−/−^ mice were divided into normal-sodium diet (NSD) and LSD groups, respectively (i.e., TASK^+/+^NSD, TASK^+/+^LSD, TASK^−/−^NSD, TASK^−/−^LSD). The basic BP, heart rate (HR) and LV structure and function parameters of each group were measured at 13 weeks of age, and the above cardiovascular parameters were measured again after 2 weeks of the LSD. To determine if an LSD could prevent the development of LV hypertrophy in TASK^−/−^ mice, 4-week-old male TASK^+/+^ and TASK^−/−^ mice were divided into four groups as well (TASK^+/+^NSD, TASK^+/+^LSD, TASK^−/−^NSD, and TASK^−/−^LSD groups). An LSD was given to TASK^−/−^LSD and TASK^+/+^LSD group mice at 4 weeks of age, and this lasted through the entire study period of 8 weeks until the mice were euthanized at 12 weeks of age. Mice in the TASK^+/+^NSD and TASK^−/−^NSD groups were fed an NSD. All animals underwent echocardiography at 4 weeks of age, and subsequently every 4 weeks thereafter, at which time the hearts were harvested for metabolomic analyses. The NSD contained 0.283% sodium, and the LSD contains 0.05% sodium (Davies et al., 2008; Guagliardo et al., 2012). All experiments were conducted under the Guide for the Care and Use of Laboratory Animals and were approved by the Animal Care and Ethical Committee of Hebei Medical University.

### 2.2 Histological analysis

The Masson trichrome staining and hematoxylin and eosin (HE) staining of heart tissues were conducted according to instructions included with the commercial kit (G1120, G1340-7, *Beijing Solarbio Science & Technology Co., Ltd.*) and as already published elsewhere (Bai et al., 2022). The mice were weighed and anesthetized, and arterial blood was taken for electrolyte and ELISA analysis. The hearts were removed and weighed, and then fixed with 4% paraformaldehyde, dehydrated, embedded in paraffin, and cut into 5-μm-thick sections. The heart weight (HW) was normalized by body weight (BW). A Panoramic Slide scanner (3D HISTECH, Budapest) was used to acquire whole-slide images and a Leica DM2000 LED microscope was used for taking high magnification images. The average myocyte cross-sectional area (MCSA) of each heart was calculated using ImageJ software from a minimum of 50 cardiomyocytes per heart. A fibrosis fractional was also measured with ImageJ and expressed as a percentage of the green area in the visual field.

### 2.3 Total RNA isolation and real-time PCR analysis

Samples from the LV were separated, flash frozen in liquid nitrogen, and stored at −80°C until RNA isolation was completed. Total RNA was extracted using the Eastep Super Total RNA Extraction Kit (LS1040, Promega) and reverse transcribed with HiScript III RT SuperMix (R323-01, Vazyme). Quantitative real-time PCR was conducted using the ChamQ Universal SYBR qPCR Master Mix (Q711-02, Vazyme). The primers were as follows: Brain natriuretic peptide (BNP), forward 5′-GAG​GTC​ACT​CCT​ATC​CTC​TGG-3, reverse 5′-GCC​ATT​TCC​TCC​GAC​TTT​TCT​C-3’; β-myosin heavy chain (βMHC), forward 5′-ACT​GTC​AAC​ACT​AAG​AGG​GTC​A-3, reverse 5′-TTG​GAT​GAT​TTG​ATC​TTC​CAG​GG-3’; and Glyceraldehyde-phosphate dehydrogenase (GAPDH): forward 5′-GCA​AAT​TCA​ACG​GCA​CAG​TCA​AGG-3′, reverse 5′-TCT​CGT​GGT​TCA​CAC​CCA​TCA​CAA-3′, and all primers in this study were purchased from *Sangon Biotech Co., Ltd* (Shanghai). The target gene expression was calculated using the 2^−ΔΔCT^ method, and the GAPDH gene level was used to normalize the expression of the target gene.

### 2.4 ELISA analysis

Whole blood was collected from the left common carotid artery, and centrifuged (1,000 g, 4°C, 15 min) to yield the plasma. Plasma samples were stored at −80°C until the day of experiment. The plasma aldosterone concentration was determined using the Aldosterone Parameter Assay Kit (KGE016, R&D Systems) according to the manufacturer’s instructions.

### 2.5 Echocardiography

The mice were anesthetized with 3% isoflurane in an induction chamber and maintained with 1.0%–1.5% isoflurane delivered via 100% O_2_ mask inhalation during echocardiography (Li et al., 2019). Transthoracic echocardiography was performed using the Vevo 2,100 high-frequency, high-resolution digital imaging system (FUJIFILM VisualSonics Inc., Toronto, Canada) with a high-frequency linear array transducer probe (MS400). All echocardiograms were performed by one trained individual. Each of these captured image loops included 10 to 20 cardiac cycles, and all echocardiogram data were averages from at least three cycles per loop. The parameters were measured from M-mode images taken from the parasternal short-axis view at the papillary muscle level. LV diastolic function was evaluated from measurements of the peak LV early filling velocity (E) and the peak left atrial filling velocity (A), and calculation of the E/A ratio.

### 2.6 BP and HR recording

The methods of telemetric recording of BP and HR in conscious, freely moving mice have been previously described in our other recent paper (Shi et al., 2019). Briefly, after induction of anesthesia in a chamber full of isoflurane for 1–2 min, mice were anesthetized with inhalational anesthesia. Anesthetic depth was confirmed by toe pinch. A transmitter (HD-X11) was placed under the skin in the abdominal area, with a pressure catheter inserted into the left common carotid artery. At least 5 days after healing, ambulatory systolic BP, diastolic BP, and HR were measured every 1 min for 24 h at 1 kHz. Computer-based acquisition hardware and software (Ponemah v6.00, Data Sciences International, United States) were used for the recording and analysis of the telemetry signals.

### 2.7 Metabolomics analysis of heart tissue

For our metabolomics analysis, the mice were first anesthetized with pentobarbital sodium (60 μg/g, i.p.). After anesthesia, the hearts were excised, washed in ice-cold PBS to remove excess blood, and snap-frozen in liquid nitrogen and maintained at −80°C until processing. The tissue samples were then thawed on ice, 1.0 mL of prechilled MeOH/H_2_O (−80°C, 1:1, v/v) was added to the tubes, and the mixture was then homogenized. Next, the mixture was centrifuged at 14,000 g for 10 min at 4°C. The supernatant was then transferred to a clean microcentrifuge tube for UPLC-HRMS analysis. Untargeted metabolomics profiling was then performed on an ultra-high performance liquid chromatography tandem high-resolution mass spectrometry (Model: UPLC: Thermo Ultimate 3,000 and HRMS: Thermo Q Exactive plus) platform at the Hebei Medical University Core Facilities and Centers. The chromatographic and mass spectrometric parameters were used referring to literature (Kamboj et al., 2021). Briefly, the chromatographic separation was attained on a Waters ACQUITY ULPL BEH C18 column (1.7 μm, 100 mm × 2.1 mm) with Mobile Phase A (10 mM ammonium acetate in water) and Mobile Phase B (acetonitrile) at a flow rate of 0.3 mL/min. All samples were analyzed in the Full MS/dd-MS2 model for both ESI positive and negative scans. Compound Discoverer 3.2 (Thermo Fisher Scientific) was used to identify metabolites from the raw data files generated by the UHPLC-HRMS. An untargeted metabolomics workflow was used to identify the differences in metabolites between samples from the three study groups. This workflow performed the retention time alignment, unknown metabolite detection, metabolite grouping across all samples, predicted elemental compositions for all metabolites, filled gaps across all samples, corrected the chemical background (using blank samples) and normalized the data by using constant mean parameters. QC samples were used for batch normalization and statistical data analysis. Identification of the metabolites was done by using mzCloud (ddMS2) and ChemSpider (formula or exact mass) along with similarity searches for all compounds with ddMS2 data using mzCloud.

### 2.8 Statistical analysis

Data were expressed as mean ± SEM. All statistical analysis was conducted using Prism (V9.0, GraphPad Prism, United States). The *t-*test was used to compare differences between two groups, and the statistical significance of multiple groups was determined by one-way ANOVA with Fisher’s LSD test or two-way ANOVA with Bonferroni’s test. *p* values < 0.05 were considered statistically significant. For untargeted metabolomic data, groups area ratios, fold change (log2 scale), Principal Component Analysis (PCA), as well as differential analysis by ANOVA were analyzed using the Compound Discoverer™ 3.2 software.

## 3 Results

### 3.1 Analysis of arterial blood gases and electrolytes in TASK^−/−^ mice

It is common for PA patients to suffer from fluid, electrolyte, and acid-base imbalances. To determine whether this characteristic also occurred in the TASK^−/−^ mice, we measured the parameters of arterial blood gases and electrolytes in this PA model. As shown in Table 1, TASK^−/−^ mice on an NSD displayed higher arterial blood Na^+^, HCO_3_^−^, pCO_2_, blood base excess, base excess of the extracellular fluid, concentration of total carbon dioxide (cTCO_2_), and lower K^+^ levels than those in TASK^+/+^ mice (*n* = 8 in TASK^+/+^ and *n* = 11 in TASK^−/−^, *p* < 0.05, Table 1). As such, normal diet TASK^−/−^ mice exhibited hypernatremia, hypokalemia and mild acid-base balance disorders despite their pH remaining in the normal range.

**TABLE 1:** Electrolytes/chemistries/blood gases.

Parameter	Normal range	TASK^+/+^ (‾X±SE, *n* = 8)	TASK^−/−^(‾X±SE, *n* = 11)	*p*-Value
pH	7.35–7.45	7.36 ± 0.01	7.38 ± 0.01	0.1383
pCO_2_(mmHg)	35–48	39.69 ± 1.43	40.05 ± 5.65	0.0357*
PO_2_(mmHg)	83–108	110.20 ± 5.36	114 ± 5.14	0.6153
cHCO_3_^-^(mmHg)	21–28	22.19 ± 0.39	26.75 ± 0.63	0.0001*
cTCO2(mmol/L)	22–29	22.51 ± 2.88	28.12 ± 2.19	0.0008*
BE(b) (mmol/L)	−2∼+3	−0.38 ± 0.28	0.83 ± 0.70	0.0003*
BE (ecf) (mmHg)	−2∼+3	−2.7 ± 0.8	1.67 ± 0.70	0.0008*
cSO_2_(%)	94–98	97.89 ± 0.31	98.13 ± 0.28	0.5720
Na^+^(mmol/L)	138–146	143.4 ± 0.6	146.4 ± 0.87	0.0179*
K^+^(mmol/L)	3.5–4.5	3.99 ± 0.19	3.01 ± 0.14	0.0005*

Analysis of the arterial blood gases, and electrolytes in TASK^**+/+**^ and TASK^**−/−**^ mice fed normal diet. The symbol * indicates a statistically significant difference between TASK^+/+^ and TASK^−/−^ mice on normal diet (unpaired *t*-test, *p* < 0.05). cSO_2_, oxygen saturation; cTCO_2_, concentration of total carbon dioxide; BE (b), base excess-blood; BE (ecf), base excess of extracellular fluid.

### 3.2 An LSD lowers BP in TASK^−/−^ mice

**FIGURE 1:**
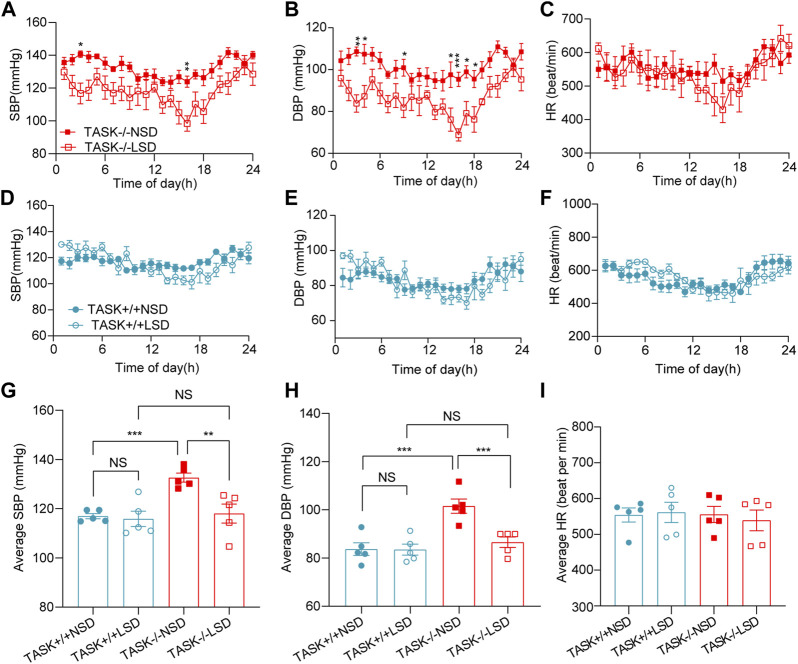
LSD lowers BP in TASK^**−/−**^ mice. SBP, DBP, and HR were monitored over 24 h in TASK^+/+^ and TASK^−/−^ mice using a telemetric BP monitoring system. 24 h-dynamic SBP, DBP, and HR measurements of TASK^−/−^ mice **(A–C)** and TASK^+/+^ mice **(D–F)** fed an NSD or LSD for 2 weeks (*n* = 5 in each group, *p < 0.05, **p < 0.01, ***p < 0.0005, two-way ANOVA with Bonferroni’s test). **(G–I)**, 24-h average SBP, DBP, and HR in the various groups (**p < 0.005, ***p < 0.001, one-way ANOVA with Fisher’s LSD test). ns, no significant difference.

High sodium environment is considered one of the major contributors to hypertension (Rucker et al., 2018). In order to determine whether high sodium levels play an important role in the development of hypertension in TASK^−/−^ mice, we measured the 24-h ambulatory systolic BP (SBP), diastolic BP (DBP), and HR using a radio-telemetry system in conscious, freely moving mice before and after 2 weeks of the LSD as described above. In line with our earlier results, TASK^−/−^ mice with an NSD were hypertensive in their 24-h ambulatory arterial pressure (Supplementary Figure S1). As shown in Figures 1A, B, the two-week LSD decreased the 24-h ambulatory SBP and DBP in TASK^−/−^ mice, and the difference was statistically significant at some time points (*n* = 5 in each group, *p* < 0.05). In contrast, TASK^+/+^ mice that were fed the LSD exhibited no significant changes in 24-h ambulatory SBP or DBP (*n* = 5 in each group, *p* > 0.05, Figures 1D, E). Furthermore, the LSD significantly reduced the 24-h average SBP (mmHg) (133 ± 2 to 118 ± 4, *p* < 0.005) and DBP (mmHg) (102 ± 3 to 87 ± 2, *p* < 0.001) in TASK^−/−^ mice but not TASK^+/+^ mice (SBP:117 ± 1 to 116 ± 3, DBP:84 ± 3 to 84 ± 2, *p* > 0.05, Figures 1G, H). Moreover, the LSD did not significantly alter the HR in either group (Figures 1C, F, I). These results show that dietary sodium restriction caused a reduction in BP in TASK^−/−^ mice, suggesting that a high sodium environment may be required for hyperaldosteronism-induced hypertension.

### 3.3 An LSD alleviates LV wall thickness in TASK^−/−^ mice

**FIGURE 2:**
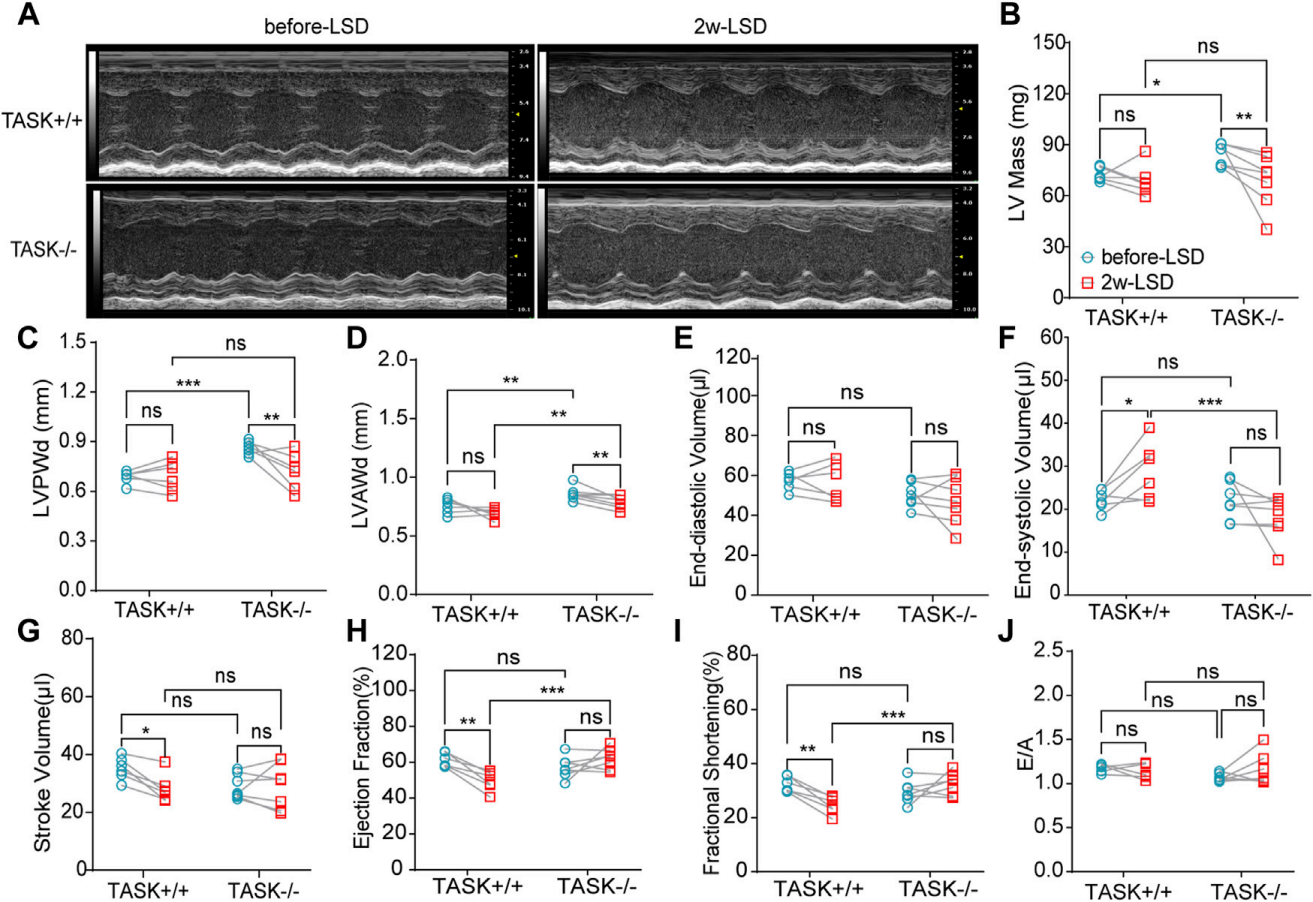
LSD alleviates LV wall thickness in TASK^**−/−**^ mice. Representative M-mode echocardiogram images of the LV from TASK^+/+^ and TASK^−/−^ mice before and after a two-week LSD **(A)**. Quantitative comparison of echocardiographic parameters including LV mass **(B)**, LVAWd **(C)**, LVPWd **(D)**, LVEDV **(E)**, LVESV **(F)**, stroke volume **(G)**, ejection fraction **(H)**, fractional shortening **(I)** and E/A **(J)** from TASK^+/+^ and TASK^−/−^ mice before and 2 weeks after LSD. The symbol * indicates a significant difference between TASK^+/+^ and TASK^−/−^ using unpaired *t*-tests, *p < 0.05, ****p < 0.0001. The symbol # indicates a significant difference between before and after 2 weeks of LSD based on paired *t*-tests, #*p* < 0.05, ##*p* < 0.005.

Early studies have found that excess aldosterone causes LV hypertrophy and that patients with PA have more LV hypertrophy than those with essential hypertension (Monticone et al., 2018; Tsai et al., 2021). To determine if cardiac hypertrophy is also present in TASK^−/−^ mice, we performed echocardiography and hemodynamic analysis to assess the geometry and function of the LV. The M model echocardiography analysis (Figure 2A) showed that adult TASK^−/−^ mice (12–13 weeks old, *n* = 6) presented greater LV mass, LV posterior wall end-diastolic thickness (LVPWd), LV anterior wall end-diastolic thickness (LVAWd), and slightly lower LV end-diastolic volume (LVEDV), and stroke volume (SV) in comparison to their TASK^+/+^ counterparts (*n* = 7, *p* < 0.05 Figures 2B–E, G). The LV end-systolic volume (LVESV), the ejection fraction (EF), fractional shortening (FS), and the E/A ratio were all not statistically different between the two genotypes (*p* > 0.05, Figures 2F, I, J). In addition, qPCR results showed that the mRNA expression levels of BNP and β-MHC in LV tissue, which are known to be upregulated in pathological hypertrophy, were significantly upregulated in TASK^−/−^ mice compared to TASK^+/+^ mice (Supplementary Figures S2A, B). Moreover, our Masson staining results showed that the fibrosis area was not statistically different between the groups either (Supplementary Figures S2C, D). Together, these data demonstrate that adult TASK^−/−^ mice with preserved heart function developed apparent pathological cardiac hypertrophy.

**FIGURE 3:**
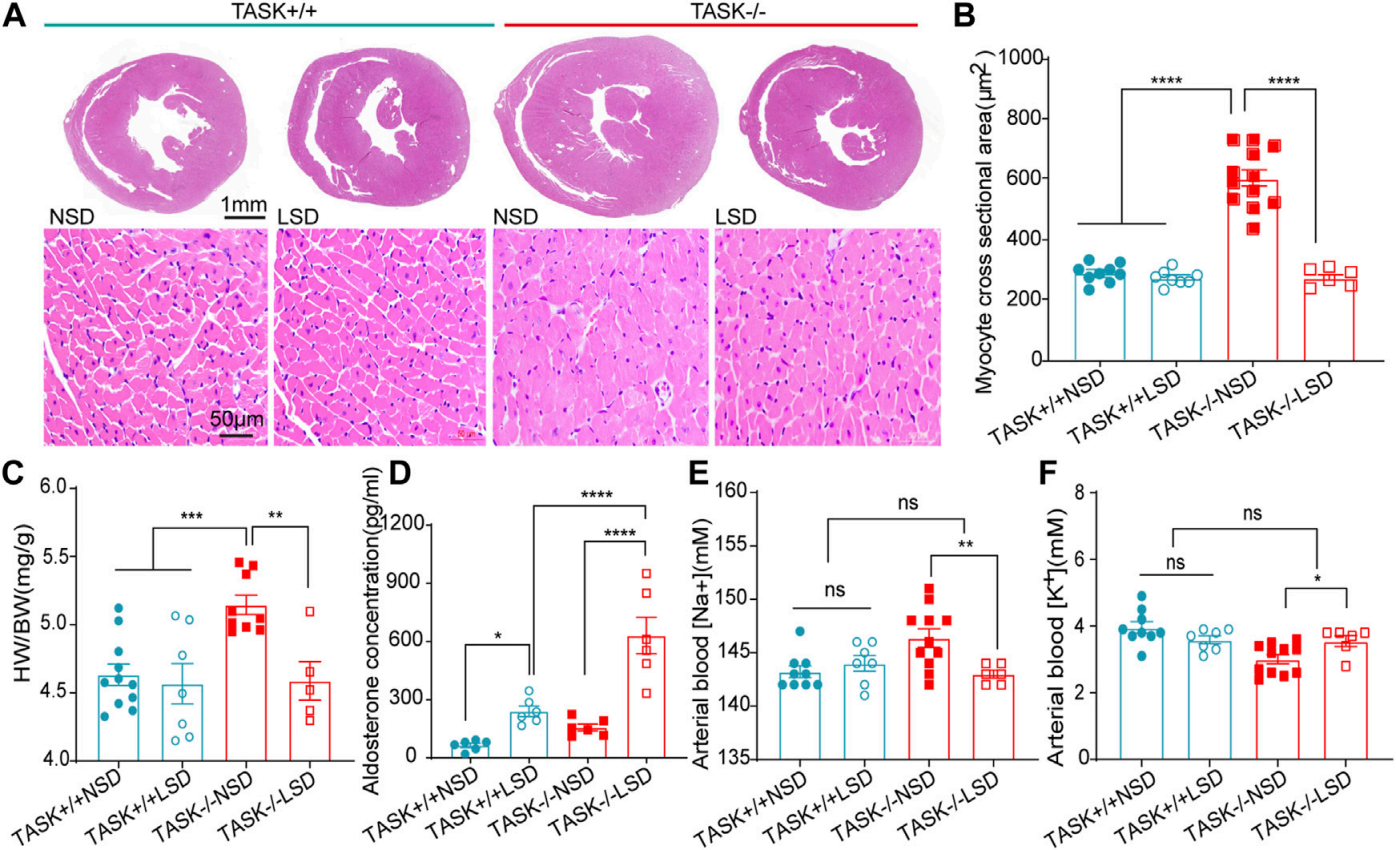
Morphological and biochemical analysis. **(A)** HE staining images of transverse heart sections at the level of the papillary muscle (top images, scale bars: 1 mm) and LV myocyte cross sections (bottom images, scale bars: 50 μm). Quantification of average MCSA (>50 myocytes per heart) in the randomly selected field **(B)**, ****p < 0.0001, one-way ANOVA with Fisher’s LSD test. The HW/BW **(C)**, plasma aldosterone concentration **(D)**, arterial blood Na^+^ **(E)** and arterial blood K^+^ **(F)** in TASK^+/+^ and TASK^−/−^ mice with NSD or LSD (*p < 0.05, **p < 0.005, ***p < 0.0005, one-way ANOVA with Fisher’s LSD test).

Following 2 weeks of LSD, the LV mass, LVPWd, and LVAWd of TASK^−/−^ mice decreased significantly (before LSD vs. after LSD, *p* < 0.05, Figures 2A–D). However, the LVEDV and LVESV, SV, EF, FS, and E/A ratio were not significantly changed (*p* > 0.05, Figures 2E–J). Additionally, 2 weeks of the LSD did not significantly affect the LV mass, LVPWd, LVAWd, or LVEDV in the TASK^+/+^ mice (*p* > 0.05, Figures 2A–E) although LVESV, SV, EF, and FS were variably altered (*p* < 0.05, Figures 2E–G). Histological analysis revealed that the MCSA was significantly higher in TASK^−/−^ mice with an NSD than that in TASK^+/+^ mice, and this difference diminished after 2 weeks of the LSD (TASK^+/+^NSD vs. TASK^−/−^NSD vs. TASK^−/−^LSD, *n* = 9 vs. *n* = 12 vs. *n* = 6, *p* < 0.001, Figures 3A, B). TASK^−/−^ mice on an NSD had a consistently significantly larger HW/BW ratio than TASK^+/+^ mice as well, a difference that was eliminated by 2 weeks of sodium restriction (TASK^+/+^NSD vs. TASK^−/−^ NSD vs. TASK^−/−^LSD, *n* = 11 vs. *n* = 9 vs. *n* = 5, Figure 3C). In addition, 2 weeks of low sodium treatment had no significant effect on HW/BW or MCAS in TASK^+/+^ mice (NSD vs. LSD, *n* = 11 vs. *n* = 7, *p* > 0.05, Figures 3B, C). Thus, it appears that higher sodium levels are a critical contributing factor to LV hypertrophy in TASK^−/−^ mice. Interestingly, ELISA analysis showed that 2 weeks of the LSD significantly enhanced plasma aldosterone concentrations (pg/mL) in both strains, but the rise was most pronounced in the TASK^−/−^ group (*n* = 6 in each group, *p* < 0.05, Figure 3D). Electrolyte analysis revealed that the dietary sodium restriction significantly reduced blood Na^+^ levels and restored blood K^+^ concentration in TASK^−/−^ mice (NSD vs. LSD, *n* = 11 vs. *n* = 6, *p* < 0.05) and that the TASK^+/+^ mice were able to adapt to maintain Na^+^ and K^+^ balance (NSD vs. LSD, *n* = 9 vs. *n* = 7, *p* > 0.05, Figures 3E, F). Hence, after 2 weeks of sodium restriction, TASK^−/−^ mice exhibited higher plasma aldosterone concentrations but nearly normal arterial blood Na^+^ and K^+^ levels. Importantly, LV hypertrophy was effectively alleviated in the TASK^−/−^ mice, though. Thus, even higher aldosterone concentrations did not result in cardiac hypertrophy in TASK^−/−^ mice with LSD.

### 3.4 An LSD prevents the development of LV hypertrophy in TASK^−/−^ mice

**FIGURE 4:**
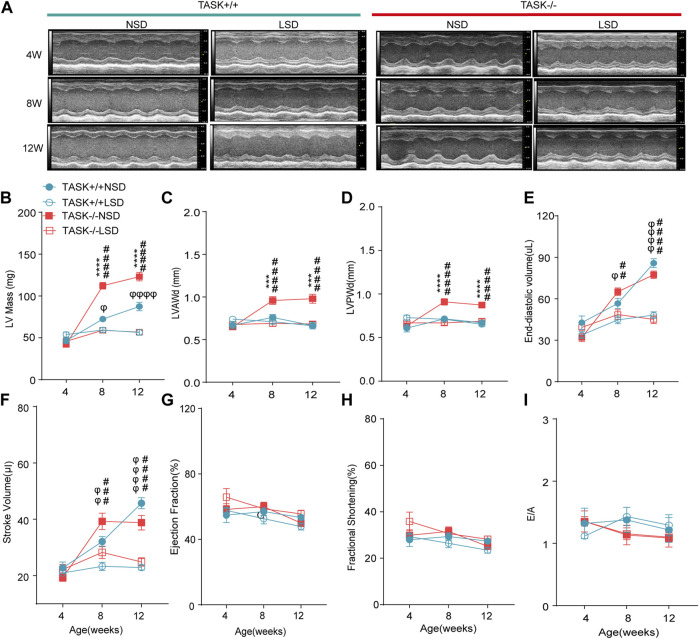
LSD prevents the development of LV hypertrophy in TASK^**−/−**^ mice. **(A)** Representative M-mode echocardiogram images of each group of mice at 4 weeks, 8 weeks, and 12 weeks of age. Quantitative comparison of echocardiographic parameter changes in different groups, including LV Mass **(B)**, LVAWd **(C)**, LVPWd **(D)**, LVESD **(E)**, stroke volume **(F)**, ejection fractions **(G)**, fractional shortening **(H)**, and E/A ratio **(I)**. Statistical significance was analyzed by two-way ANOVA with Bonferroni’s multiple comparison test (*n* = 6 mice for each group, *p* < 0.05). The symbol * marks a comparison between TASK^+/+^ and TASK^−/−^ mice on a normal diet, and Ф and # represent a comparison between NSD and LSD in TASK^+/+^ and TASK^−/−^ mice respectively.

The progression of LV hypertrophy in TASK^−/−^ mice over time was evaluated by echocardiography at 4, 8, and 12 weeks of age. At 4 weeks of age, no significant difference in the LV morphological or functional parameters was observed between the two genotypes with a normal diet (Figure 4). At 8 and 12 weeks of age, however, the TASK^−/−^ mice had significantly higher LV mass and LVPWd than the TASK^+/+^ mice (*n* = 6 in TASK^−/−^ mice vs. *n* = 7 in TASK^+/+^ mice, *p* < 0.001, Figures 4A–D). In addition, no significant differences were observed in the LVEDV, SV or any other functional parameters, such as EF, FS, or E/A ratio (*p* > 0.05, Figures 4E–I). Those results indicate that TASK^−/−^ mice with a normal diet show spontaneous LV hypertrophy as they aged.

To test the hypothesis that aldosterone-induced cardiac injury only occurs in the presence of an inappropriate salt state. We fed TASK^−/−^ and TASK^+/+^ mice an LSD from the age of 4 weeks, and the morphological and functional parameters of the LV were assessed at 4, 8, and 12 weeks of age. Compared to the NSD group, mice fed an LSD showed a lower LV mass, LVAWd, LVPWd, LVEDV, and SV at 8 and 12 weeks old in the TASK^−/−^ group (*n* = 6 in each group, *p* < 0.005, Figures 4A–F). In contrast, TASK^+/+^ mice consumed the LSD had no differences in LVAWd or LVPWd compared to the NSD group (*p* > 0.05, Figures 4C, D) but showed significant reductions in LV mass, LVEDV, and SV at 8 and 12 weeks old (NSD vs. LSD, *n* = 6 vs. *n* = 7, *p* < 0.05, Figures 4B, E, F). In addition, neither TASK^+/+^ nor TASK^−/−^ mice manifested a significant difference in EF, FS, or E/A ratio between the two different kinds of diet (*p* > 0.05, Figures 4G–I). All of these parameters were not statistically different between TASK^+/+^ and TASK^−/−^ mice on the LSD, however. Collectively, an LSD prevented the development of LV hypertrophy in TASK^−/−^ mice, and these findings supported that an inappropriate salt state contributed to aldosterone-induced cardiac injury.

### 3.5 Effect of the LSD on metabolite profiling in TASK^−/−^ mouse hearts

**FIGURE 5:**
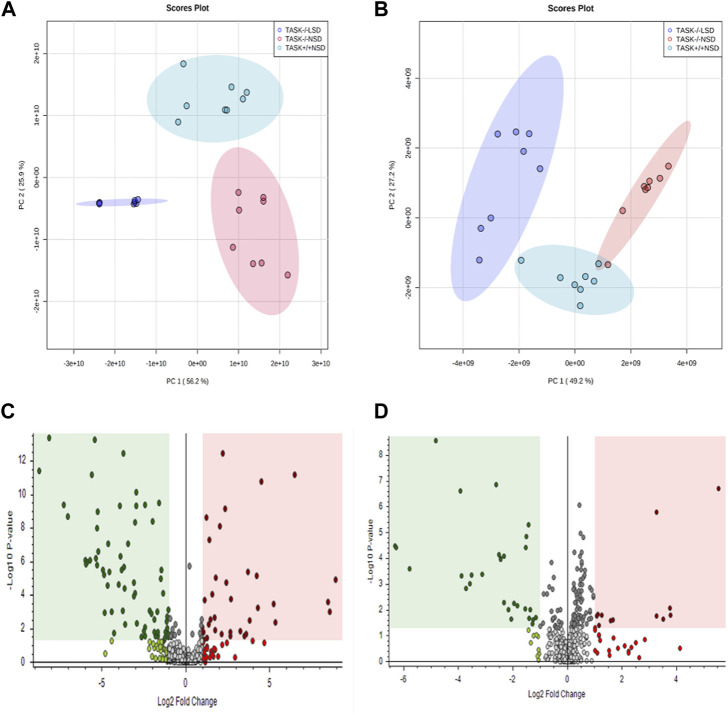
Principal component analysis and volcano plots of detected metabolites in positive mode and negative mode. PCA scores were plotted using positive **(A)** and negative **(B)** ion mode datasets for the TASK^+/+^-NSD, TASK^−/−^-NSD, and TASK^−/−^-LSD groups. PC1, Principal component 1; PC2, Principal component 2. The volcano plots show the differential expression of metabolites between the TASK^+/+^-NSD and TASK^−/−^-NSD groups under positive **(C)** and negative **(D)** ion mode. Log_2_^Fold change^ is shown for TASK^−/−^-NSD versus TASK^+/+^-NSD. The pink areas represent significantly upregulated metabolites, and the green areas indicate significantly downregulated metabolites, based on Log_2_^Fold change^ and *p*-values <0.05.

**FIGURE 6:**
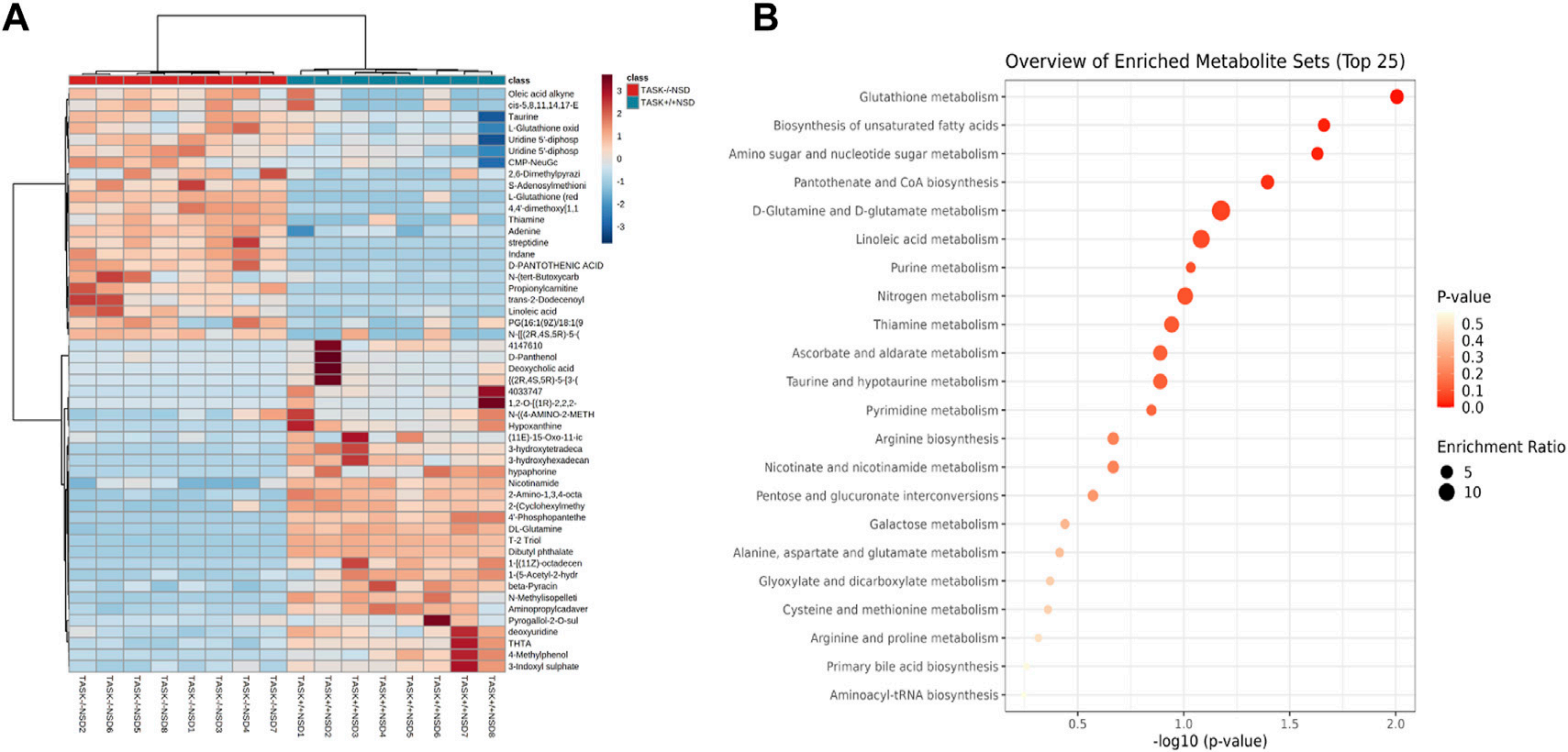
Enrichment analysis of significantly changed metabolites. Heatmap visualization of 51 differential metabolites between the TASK^+/+^ and TASK^−/−^ mice with a normal diet **(A)**. Comparing TASK^−/−^ to TASK^+/+^ mice, 22 of their metabolites were upregulated, while 29 were downregulated (Wilcoxon rank-sum test, FDR-corrected *p* < 0.05). A heatmap was generated using Pearson’s chi-squared test for distance measurement and the Ward test for the clustering algorithm. Colors on the heatmap indicate expression levels (red means high; blue means low). **(B)** The bubble plot displays metabolic pathway enrichment analysis of significantly changed metabolites from **(A)**. The metabolic pathway was analyzed with MetaboAnalyst 5.0 using KEGG. A bubble’s size indicates its enrichment ratio. Colors indicate the significance of the annotated enrichment value based on a −log10 (adjusted *p*-value).

Most cardiovascular diseases are associated with metabolic disturbances. In order to explore the mechanisms underlying the hypertrophic change, we conducted an untargeted metabolomics analysis on the heart tissue of adult TASK^+/+^ mice fed an NSD and TASK^−/−^ mice with either an NSD or LSD. The results of UPLC-HRMS analysis performed with Compound Discoverer 3.2 showed that among 1,006 putative metabolites, 339 compounds matched with those in the mzCloud (match score >60), Metabolika Search, and Chemspider database after combining the data in positive and negative modes (Supplementary Table S1). PCA was conducted to investigate the distribution and tendency of these three groups. As illustrated in Figure 5, there was a good separation pattern among the groups in both positive (Figure 5A) and negative (Figure 5B) ion mode, indicating that metabolic differences were evident among the three groups. To investigate metabolic differences between TASK^+/+^ and TASK^−/−^ mouse hearts, we identified regulated metabolites using a log_2_^Fold change^ > 1 and an adjusted *p*-value of <0.05. A total of 51 differential metabolites (Supplementary Table S2) found by this method in both positive and negative mode are summarized by volcano plots (Figures 5C, D) and heatmaps (Figure 6A). There were 22 upregulated metabolites and 29 down-regulated in TASK^−/−^ mice compared to TASK^+/+^ mice (*p* < 0.05, Wilcoxon rank-sum test, Figure 6A; Supplementary Table S3). Among these significantly regulated metabolites, 26 compounds are well annotated in KEGG databases (Supplementary Table S3). Investigating the possible metabolic pathways further, all 26 discriminant metabolites were imported into MetaboAnalyst 5.0 (https://www.metaboanalyst.ca/) for pathway and enrichment analysis using the KEGG database. Figure 6B shows the top 5 enriched metabolic pathways that were likely to have been involved in the pathological cardiac hypertrophy of TASK^−/−^ mice, including Glutathione metabolism; biosynthesis of unsaturated fatty acids; amino sugar and nucleotide sugar metabolism; pantothenate and CoA biosynthesis; D-glutamine and D-glutamate metabolism; and linoleic acid metabolism.

**FIGURE 7:**
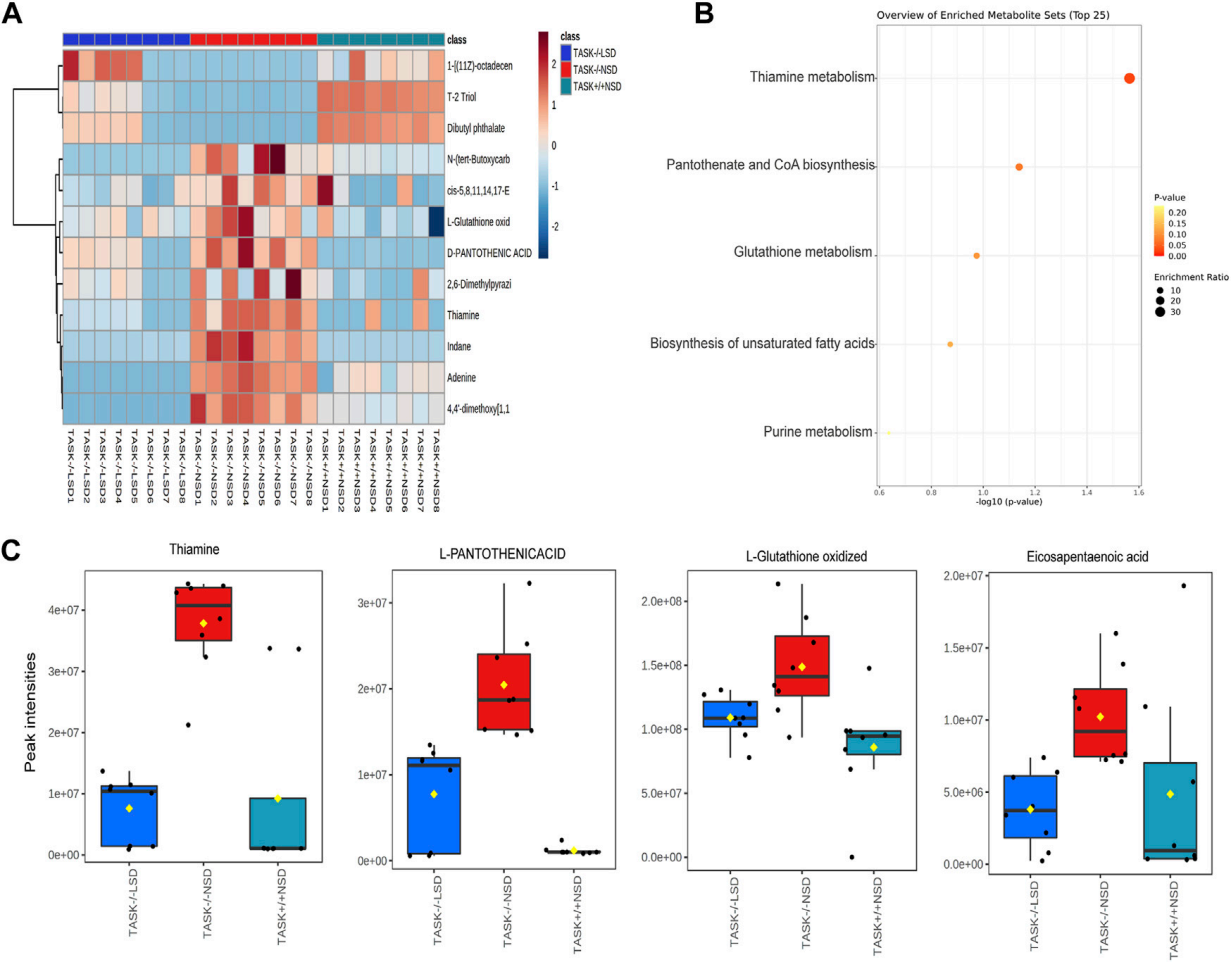
Metabolites that were significantly altered in TASK^−/−^ mice and prevented by sodium restriction. **(A)** Heat maps summarize the metabolites significantly regulated in TASK^−/−^ mice compared to TASK^+/+^ mice, and which were reversed by the two-week LSD (*n* = 8 in each group, *p* < 0.05, one-way ANOVA with Fisher’s LSD test). A heatmap was generated using Pearson’s chi-squared test for distance measurement and the Ward test for the clustering algorithm. Colors on the heatmap indicate expression levels (red means high; blue means low). **(B)** The bubble plot displays metabolic pathway enrichment analysis of those significantly reverted metabolites in response to the LSD from **(A)**. Box-and-whisker plots summarize the normalized values **(C)**. The Box-and-whisker plots include a central box that shows the interquartile range and a median line inside the box. Normal diet TASK^+/+^ mice vs. normal diet TASK^−/−^ mice vs. LSD TASK^−/−^ mice, *p* < 0.05, one-way ANOVA with Fisher’s LSD test.

In exploring the role of hypernatremia in myocardial metabolic disorders in the TASK^−/−^ mice, we hypothesized that these metabolites were significantly altered in TASK^−/−^ mice and that their expression should be reversed by an LSD. Interestingly, we found that 12 of these metabolites, which were upregulated or downregulated in the TASK^−/−^ group compared to the TASK^+/+^ group, were significantly reversed by the two-week LSD (*p* < 0.05, one-way ANOVA with Fisher’s LSD test, Figure 7A, Supplementary Table S5). The expression of metabolites such as oxidized L-Glutathione, cis-5,8,14,17-Eicosapentaenoic acid, D-Pantothenic acid, thiamine and adenine were upregulated in TASK^−/−^ mice and reversed by sodium restriction (Figures 7A–C). Furthermore, enrichment analysis using reversible metabolites showed that these pathways were thiamine metabolism, pantothenate and CoA biosynthesis, glutathione metabolism, biosynthesis of unsaturated fatty acids, and purine metabolism (Figure 7B). Based on our metabolomics analysis, some of the disturbances in heart metabolism in TASK^−/−^ mice can be reversed by an LSD may contribute to the development of pathological hypertrophy.

## 4 Discussion

In the present study, we explored the biochemical and cardiovascular phenotypes of TASK^−/−^ mice. Our mouse model of PA exhibited hyperaldosteronism, hypernatremia, hypokalemia, acid-base imbalance, hypertension, and LV hypertrophy, and also demonstrated that the symptoms of hypertension and LV hypertrophy in TASK^−/−^ mice were alleviated by an LSD. Moreover, hyperaldosteronism-associated cardiac hypertrophy was predicted to correlate with metabolic disturbance, and an LSD was found to prevent some of the altered metabolic pathways.

### 4.1 Blood electrolytes and cardiovascular features in TASK^−/−^ mice

Our current work refines the TASK^−/−^ mouse model of Davies et al. (2008). First, our arterial blood gas and electrolyte analysis revealed that the concentrations of Na^+^, HCO_3_^−^, BE, and cTCO_2_ were higher in TASK^−/−^ mice than in TASK^+/+^ mice, and that the concentrations of K^+^ were lower, indicating that TASK^−/−^ mice suffered from electrolyte disturbances and mild acid-base balance disorder. Metabolic alkalosis, an electrolyte disorder, is defined as a blood arterial pH > 7.43 and bicarbonate (HCO3-) >26 mmol/L (Gillion et al., 2019). Although our TASK^−/−^ mice had pH values <7.43, they were alkaline than the TASK^+/+^ mice. Over-secretion of aldosterone, resulting in enhanced Na⁺ reabsorption and K^+^ and H^+^ excretion, leads to hypokalemia and metabolic alkalosis in PA patients. However, Davies et al. established that TASK^−/−^ mice were able to achieve Na^+^ and K^+^ balance (Davies et al., 2008). This disparity in results might be due to differences in quantitative methods. In our study, we measured the amount of Na^+^ and K^+^ in arterial blood using an epoc^®^ Blood Analysis System, and Davies et al. measured it in 24-h urine by flame photometry. Second, we found that LV hypertrophy increased with aging in TASK^−/−^ mice. Indeed, clinical studies have demonstrated that PA patients have higher LV mass index, wall thickness, and more LV hypertrophy than essential hypertension control (Tsai et al., 2021). In rats with deoxycorticosterone acetate (DOCA)-salt hypertension, a classical animal model for human PA, cardiocyte hypertrophy is accompanied by cell loss and expansion of sarcoplasm with compensated LV function (Tomanek and Barlow, 1990). In line with this, our adult TASK^−/−^ mice with preserved heart function developed significantly increased LV wall thickness and LV mass. In addition, both hypertension and LV hypertrophy in TASK^−/−^ mice were mitigated by sodium restriction. However, the use of the TASK^−/−^ model has limitations. Since the TASK-1 and TASK-3 channels are widely expressed in the central nervous system and the periphery, global genetic deletion of these channels in TASK^−/−^ mice may lead to potential adverse effects (Decher et al., 2011; Guagliardo et al., 2019). Therefore, more functional experimental studies are needed to evaluate the overall accuracy of this model in the future.

### 4.2 Low sodium intake reduces hypertension in TASK^−/−^ mice

Over the past decade, clinical studies have shown that dietary sodium restriction can improve the non-dipper BP pattern of patients with PA (Uzu et al., 1998; Kimura et al., 2000; Takakuwa et al., 2002). Correspondingly, Christian et al. reported that higher sodium intake was correlated with daily doses of antihypertensive medications for BP control in PA patients (Adolf et al., 2020), and animal studies also demonstrated that excess aldosterone did not cause hypertension in the context of a low salt diet (Brilla and Weber, 1992b; Hattori et al., 2014). In line with these findings, we found that 2 weeks of an LSD could lower BP in TASK^−/−^ mice but not in control mice. While not surprising, it was noteworthy that the plasma levels of aldosterone in TASK^−/−^ mice increased several times after the two-week LSD and that their arterial sodium levels returned near to normal. Together, these findings suggest that aldosterone excess alone is not sufficient to trigger an increase in BP. However, the interactive mechanisms that underpin this finding are still not well understood. One proposed mechanism is that aldosterone and sodium have a synergistically amplifying effect. Evidence suggests that increased NaCl levels contribute to sympathetic excitability and hypertension in DOCA-salt rats due to the augmented NaCl signals by DOCA and that even a 1% decrease in plasma NaCl results in a 6% reduction in lumbar sympathetic activity (O'Donaughy and Brooks, 2006). However, due to the complexity of hypertension pathophysiology, other mechanisms may also be involved and should be investigated further.

### 4.3 Low sodium intake inhibits the development of LV hypertrophy in TASK^−/−^ mice

In a clinical study of 48 patients with PA, 75% showed concentric hypertrophy (Ori et al., 2013). In the present study, we found that adult normal diet TASK^−/−^ mice showed increased LV mass, thickened wall, and decreased LVEDV, which were ameliorated by a low-sodium intake. The interaction between circulating aldosterone and salt is critical to the pathophysiology of LV hypertrophy in PA, and this has been borne out by a series of clinical and animal studies (Funder, 2015). In PA patients undergoing surgery or medical treatment, the degree of LV reverse hypertrophy has been found to be directly and independently correlated with changes in urinary sodium excretion (Catena et al., 2016). According to these studies, the deleterious cardiovascular effects caused by elevated aldosterone require a high-salt environment. Consistent with this, our results showed that 2 weeks of an LSD could effectively reduce LV wall thickness in adult TASK^−/−^ mice. By switching to an LSD from 4 weeks of age, TASK^−/−^ mice at 8–12 weeks were protected from LV hypertrophy. The sodium content in the LSD was 0.05%, in line with previous studies (Davies et al., 2008; Guagliardo et al., 2012; Hattori et al., 2014), which is the minimum amount of sodium needed to maintain growth (Grunert et al., 1950). We found that the serum Na^+^ and K^+^ were restored to approximately normal levels in TASK^−/−^ mice fed an LSD, with aldosterone concentrations increasing several times. However, LV hypertrophy did not occur in these TASK^−/−^ mice. These results consolidate the notion that cardiac function deterioration induced by excessive aldosterone is largely dependent on salt status. Recently, Christa et al. found that PA patients showed treatment-reversible significantly higher tissue sodium signals in the myocardium, calf muscle, and skin compared to healthy controls (Christa et al., 2019; Christa et al., 2022). This is a novel explanation for the relationship between sodium and LV hypertrophy in PA. BNP is a neurohormone that is activated by volume expansion and pressure overload of cardiac ventricles (Epshteyn et al., 2003), In the present study, our results show that TASK^−/−^ mice exhibit obvious sodium retention and a rise in BNP and β-MHC mRNA levels in LV tissue. We cannot rule out the effect of volume expansion due to water-sodium retention on LV hypertrophy in TASK^−/−^ mice. Further research is therefore needed in order to explore the mechanism of hyperaldosteronism-mediated LV hypertrophy.

### 4.4 Cardiac metabolism changes in TASK^−/−^ mice

In recent decades, much effort has been exerted to improve our understanding of PA. Early studies found that the differential metabolites between PA and essential hypertension patients were enriched in several pathways involved in central carbon metabolism, amino acid metabolism, ABC transporters, and purine nucleosides indicating that PA might have a broad effect on human metabolism (Spyroglou et al., 2021; Chen et al., 2022). In the current study, we compared the abundance of cardiac metabolites in TASK^−/−^ and TASK^+/+^ mice using untargeted metabolomics, and found that the differential metabolites were enriched in pathways involved in glutathione metabolism; biosynthesis of unsaturated fatty acids; amino sugar and nucleotide sugar metabolism; pantothenate and CoA biosynthesis; and D-glutamine and D-glutamate metabolism. Most of these metabolites have been found to be associated with cardiovascular diseases. Glutamine is the most abundant non-essential amino acid in the human body, and a large portion of glutamine in cardiac metabolism is utilized for energy production (Ritterhoff et al., 2020). However, the role of glutamine metabolism in maintaining the structure and function of cardiomyocytes is largely unknown. Numerous studies suggest that glutamine protects the myocardium under diverse pathological conditions, such as ischemia and reperfusion, sepsis-induced myocardial injury, cardiomyopathy, and heart failure (Shen et al., 2021). In the present study, we found that D-glutamine and L-glutamine, components of D-glutamine and D-glutamate metabolism, were significantly downregulated in the TASK^−/−^ mice. Thus, amino acid metabolism might be a mechanism underlying PA-mediated cardiovascular risks. Oxidative stress and inflammation have been recognized as important contributors to the risk of chronic non-communicable diseases. Polyunsaturated fatty acids may regulate the antioxidant signaling pathway and modulate inflammatory processes (Djuricic and Calder, 2021). Our results show that the production of linoleic acid, alpha-linolenic acid, and cis-5,8,11,14,17-eicosapentaenoic acid involved in the biosynthesis of unsaturated fatty acid pathways were upregulated in the TASK^−/−^ mice. We have also observed that glutathione metabolism in TASK^−/−^ mice was interrupted by L-glutathione oxidation and that L-glutathione (reduction) was upregulated and aminopropyl cadaverine downregulated. It is therefore possible that the loss of redox homeostasis of these molecules may negatively impact the heart of TASK^−/−^ mice. Indeed, a significant change in the concentration of glutathione and/or its oxidation state is reported to be associated with cardiovascular disease development and progression (Senoner and Dichtl, 2019). In addition, our findings revealed that both vitamin B1 and B5 levels were elevated in the LV muscle tissue of TASK^−/−^ mice. B vitamins are precursors of essential coenzymes that play an essential role in energy metabolism (Piquereau et al., 2021). Despite vitamin B5 (pantothenic acid) being vital for CoA biosynthesis, and consequently for metabolism, research on B5 deficiency and supplementation in the context of heart disease is scarce (Piquereau et al., 2021). Several animal studies have reported that B5 has a cardioprotective effect due to its antioxidant properties (Piquereau et al., 2021). The prevalence of thiamin (vitamin B1) deficiency in heart failure patients varies from 3% to 91% and has been linked to a wide range of cardiovascular disorders (Kwok et al., 1992).

Although we have observed abnormal myocardial metabolism in TASK^−/−^ mice, the underlying mechanism remains unclear. These changes may be caused by the direct effect of excessive aldosterone on the MRs of myocardial cells or indirectly through secondary hypernatremia and hypertension. For example, metabolomics studies have demonstrated that aldosterone is associated with derivatives of the linoleic acid metabolism pathway (van der Heijden et al., 2020). High sodium diet can cause abnormal myocardial energy metabolism in the Dahl salt-sensitive rat (Kato et al., 2010). Indeed, we found that some of the altered metabolites involved in thiamine metabolism, pantothenate and Co biosynthesis, glutathione metabolism, biosynthesis of unsaturated fatty acids, and purine metabolism in TASK^−/−^ mice were prevented by the LSD. Therefore, it appears that myocardial metabolic disorders may be involved in the development of cardiac hypertrophy in TASK^−/−^ mice and that high sodium environment may play a role in this process. Further investigations are warranted to gain improved insight into its specific metabolic targets and the underlying mechanisms.

## 5 Conclusion

In conclusion, we found that adult TASK^−/−^ mice spontaneously developed hypertension and LV hypertrophy that was attenuated by low sodium intake. These observations provide evidence that the deleterious cardiovascular effects caused by excessive aldosterone require a high-salt environment. In addition, our data provide information on heart structure, function, and metabolite profiles in TASK^−/−^ mice. This mouse line may be a useful tool for exploring the pathological mechanisms of cardiovascular diseases associated with hyperaldosteronism in the future.

## Data Availability

The original contributions presented in the study are included in the article/Supplementary Materials, further inquiries can be directed to the corresponding authors.
