# Supplementary material for: Low sodium intake ameliorates hypertension and left ventricular hypertrophy in mice with primary aldosteronism
Source: Front Physiol. 2023 Feb 15;14:1136574. doi: 10.3389/fphys.2023.1136574 (PMC9974669; doi:10.3389/fphys.2023.1136574)
Supplement: Supplementary file 5 [file DataSheet1.DOCX]

Supplementary Material

Low sodium intake ameliorates hypertension and left ventricular hypertrophy in mice with primary aldosteronism

Zitian Wang^1†^, Xue Zhao^1,2†^, Lifang Bu ^1,2^, Kun Liu^5^, Ziping Li^1^, Huaxing Zhang^2^, Xiaoguang Zhang^2^, Fang Yuan^1,3^, Sheng Wang^1,3^, Zan Guo^4*^ and Luo Shi^1,3*^

*** Correspondence:** Dr. Luo Shi, Department of Neurobiology, Hebei Medical University, 361 East Zhongshan Road, Shijiazhuang, Hebei Province, China. Email: shiluo@hebmu.edu.cn; Dr. Zan Guo, Department of Physiology, Hebei Medical University, 361 East Zhongshan Road, Shijiazhuang, Hebei Province, China. Email: phyguozan18@126.com.

# Supplementary Figures and Tables

## Supplementary Figures

**
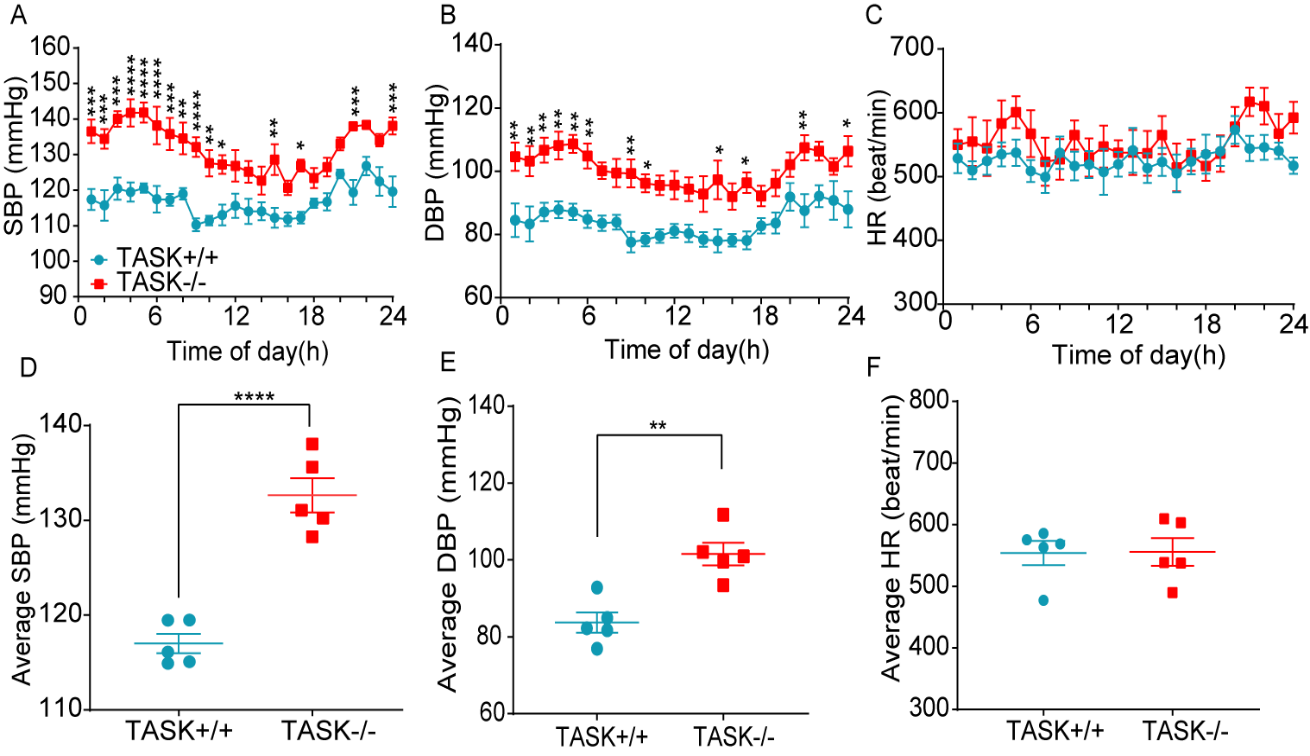
**

**Supplementary Figure 1.** Hypertension in TASK^-/-^ mice. 24h-dynamic SBP(**A**) and DBP(**B**) were significantly higher at most time points in TASK^-/-^ mice than in TASK^+/+^ mice, and HR (**C**) measured over 24 h was not different between the two mice lines at any time points (n=5 in each group, *P<0.05, ***P<0.0005, ****P<0.0001, two-way ANOVA with Bonferroni’s multiple comparisons test). (**D-F**) 24h average SBP, DBP, and HR (**P<0.005, ****p < 0.0001 as indicated, unpaired *t*-test).

**
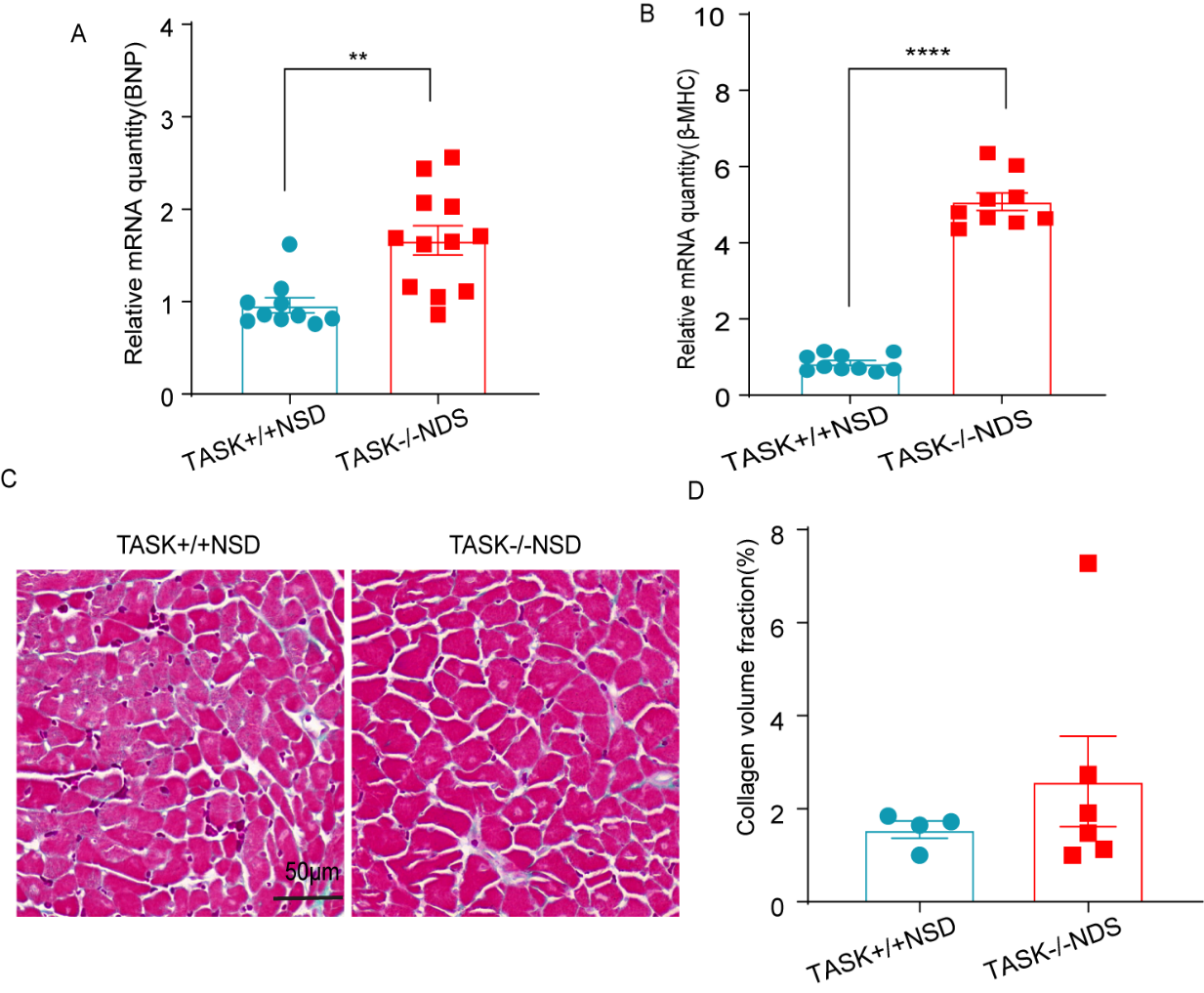
**

**Supplementary Figure 2.** Pathological left ventricular hypertrophy in TASK^-/-^ mice. (A-B) The mRNA expression levels of BNP and β-MHC in two groups (n=10 in TASK^+/+^ group and n=12 in TASK^-/-^ group, **P<0.005, ****P<0.0001, unpaired *t-*test). (C) Representative images of Masson trichrome staining in adult TASK^-/-^ (n=6) and TASK^+/+^ (n=4) mice with a normal diet (scale bars: 50 μm). (D) Collagen deposition was quantitatively analyzed as collagen volume fraction % (collagen area (blue)/total area x 100%]. No statistically significant differences were observed between the phenotypes (P>0.05, unpaired *t*-test). BNP, brain natriuretic peptide; β-MHC, beta-myosin heavy chain.
